# Repurposing Cardiac Glycosides: Drugs for Heart Failure Surmounting Viruses

**DOI:** 10.3390/molecules26185627

**Published:** 2021-09-16

**Authors:** Jan Škubník, Jiří Bejček, Vladimíra Svobodová Pavlíčková, Silvie Rimpelová

**Affiliations:** Department of Biochemistry and Microbiology, University of Chemistry and Technology Prague, Technická 3, 16628 Prague, Czech Republic; jan.skubnik@vscht.cz (J.Š.); jiri.bejcek@vscht.cz (J.B.); vladimira.pavlickova@vscht.cz (V.S.P.)

**Keywords:** cardiac steroids, digoxin, digitoxin, ouabain, lanatoside C, COVID-19, virus entry, Na^+^/K^+^-ATPase, drug repurposing, coronavirus

## Abstract

Drug repositioning is a successful approach in medicinal research. It significantly simplifies the long-term process of clinical drug evaluation, since the drug being tested has already been approved for another condition. One example of drug repositioning involves cardiac glycosides (CGs), which have, for a long time, been used in heart medicine. Moreover, it has been known for decades that CGs also have great potential in cancer treatment and, thus, many clinical trials now evaluate their anticancer potential. Interestingly, heart failure and cancer are not the only conditions for which CGs could be effectively used. In recent years, the antiviral potential of CGs has been extensively studied, and with the ongoing SARS-CoV-2 pandemic, this interest in CGs has increased even more. Therefore, here, we present CGs as potent and promising antiviral compounds, which can interfere with almost any steps of the viral life cycle, except for the viral attachment to a host cell. In this review article, we summarize the reported data on this hot topic and discuss the mechanisms of antiviral action of CGs, with reference to the particular viral life cycle phase they interfere with.

## 1. Introduction

In drug development, there are many cases in which drugs originally tested for one condition are approved for another condition, or the spectrum is expanded. Thus far, the best-known example is Viagra, which was originally developed to treat angina pectoris, but became famous and is most used to improve erectile function. However, the same substance is marketed not only as Viagra, but also as Revatio—a drug for the treatment of pulmonary hypertension [1]. Another example of a successful drug repurposing is thalidomide, which began as an antiemetic, but was soon withdrawn from the market due to its teratogenicity. However, its benefits in the treatment of *Erythema nodosum leprosum*, an immune complication in leprosy, were soon discovered [2]. Yet, thalidomide may end up treating another condition. Recent reports document its potential in inhibiting angiogenesis; therefore, it could possibly be used in cancer treatment as well [3,4]. A third example of a multifunctional drug is aspirin, used as an analgesic and antipyretic, but also as a drug to prevent blood clotting [5]. Additionally, similarly to thalidomide, more reports on aspirin antitumor activity have recently emerged [6].

Another example of drug repositioning is represented by cardiac glycosides (CGs); they have been used for several decades as drugs to treat heart failure and arrhythmias. Their mechanisms of action are mainly connected to the inhibition of Na^+^/K^+^-ATPase (NKA), which regulates the intracellular concentration of sodium and potassium ions and, in dependence on that, also of calcium ions. These changes in the intracellular concentration of Ca^2+^ result in a positive inotropic effect on the heart muscle, for which they are used in the aforementioned indications [7,8]. Interestingly, in the second half of the last century, researchers discovered that CGs have antitumor effects on various cancer types [9]. These effects were formerly thought to be mediated by changes in concentrations of the intracellular Ca^2+^, which also serve as apoptosis mediators. Currently, however, anticancer activity is mainly attributed to the interaction of CGs with NKA as a receptor part of a large signalosome, which comprises various cellular pathways [10,11,12,13,14,15]. Therefore, the spectrum of CG actions might be much broader. This was indeed confirmed during the last few years when it was reported that CGs also have antiviral effects through interaction with NKA. In this review article, we provide an overview of the data on the antiviral activity of CGs, supporting the importance of these compounds in antiviral therapy.

## 2. Cardiac Glycosides and Their Target

CGs are natural steroid compounds originally isolated from *Digitalis* sp. Their beneficial cardiotonic properties were utilized centuries ago, and were first extensively described by William Withering in 1785, who systematically used *Digitalis purpurea* extracts for the treatment of heart disorders. CGs do not occur only in *Digitalis* sp., but also in other plants (*Nerium oleander* [16], *Thevetia peruviana* [17], or *Convallaria majalis* [18]). Moreover, they occur in animals, mainly in toads [19], fireflies [20], or in monarch butterflies (*Danaus plexippus*), the larvae of which sequester CGs from milkweed and use it as a defense against avian predators, in which they cause vomiting [21].

**Figure 1:**
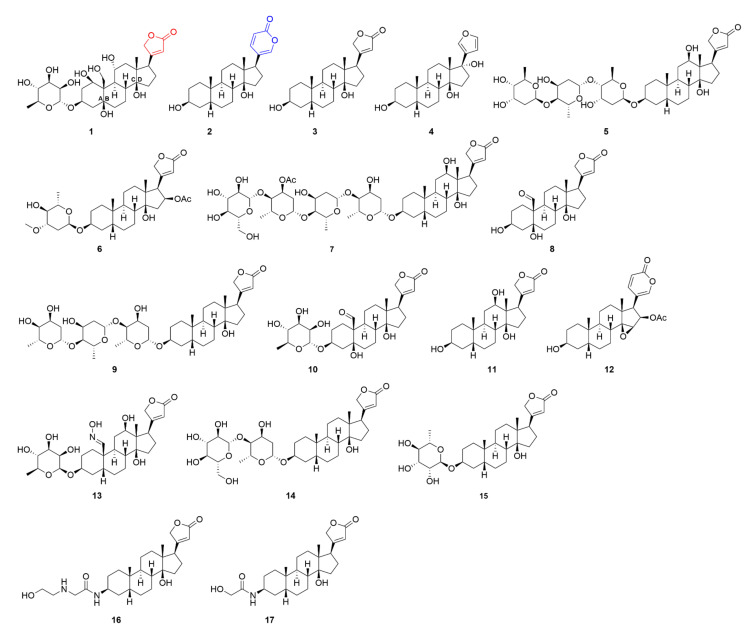
Chemical structures of cardenolide ouabain (**1**), bufadienolide bufalin (**2**), digitoxigenin (**3**) and its derivative rostafuroxin (**4**), digoxin (**5**), oleandrin (**6**), lanatoside C (**7**), strophanthidin (**8**), digitoxin (**9**), convallatoxin (**10**), digoxigenin (**11**), cinobufagin (**12**), a semisynthetic cardenolide RIDK-34 (**13**), glucoevatromonoside (**14**), evomonoside (**15**), C10 (**16**), and C11 (**17**). Compound **1** has a five-membered lactone ring (in red) and compound **2** has a six-membered lactone ring (in blue).

Besides their origin, CGs can be divided by their chemical structures, into two main groups—cardenolides and bufadienolides (Figure 1). The structure of CGs generally consists of a steroid core, sugar moiety, and a lactone ring. Cardenolides have a five-membered lactone ring and occur mainly, but not only, in plants, whereas bufadienolides contain a six-membered lactone ring and are present predominantly in animals. The steroid core is conserved in all CGs, and in contrast to human corticosteroids or sex hormones, the B and C rings are in *trans*-conformation, and A/B and C/D rings are *cis*-oriented [22]. Great structural variability is ensured by the connected sugars, the number and structure of which differs in every CG. CGs contain very common sugars, such as mannose, glucose, or galactose, and highly specific ones, such as digitoxose, thevetose, or cymarose [23]. The complex structure of CGs significantly affects their biological properties, which, as aforementioned, are connected with binding to NKA.

NKA is a transmembrane protein with enzyme activity responsible for pumping K^+^ ions inside cells and Na^+^ ions outside cells. The energy for this process is gained from ATP hydrolysis. Given this ion pumping activity, NKA is crucial for maintaining stable membrane potential and, thus, it directly affects the right functioning of the cells. As for the structure, NKA is composed of α and β subunits, which naturally form a dimer. The α subunit is the catalytic part, which is responsible for the whole enzyme activity, while the β subunit is involved in the translation of the α subunit and also impacts the binding affinity of Na^+^ and K^+^ ions. Both subunits occur in four and three tissue-specific isoforms, respectively. For an extensive review on NKA, see reference [24].

As aforementioned, NKA not only has the pumping function, it also plays a vital role in cellular signaling, as it is a key member of the complex signalosome. NKA signalosome includes many crucial receptors, the most important of which is the epidermal growth factor receptor (EGFR), which is further linked, for example, to Ras/Raf/MEK/ERK (rat sarcoma protein/rapidly accelerated fibrosarcoma protein/mitogen-activated protein kinase kinase/extracellular signal-regulated kinase) signaling cascade [25]. This cascade is involved in the regulation of the cell cycle and apoptosis [26]. Therefore, NKA is an ideal therapeutic target, for example, in cancer treatment, and CGs, as natural NKA effectors, have great medicinal potential. Nevertheless, altering the function of NKA is not the only mechanism of the CG action. For example, CGs were shown to bind nuclear receptors, particularly transcription factors, and suppress the immune system and carcinogenesis, or disrupt hormonal balance [27]. Hormonal processes—besides binding to transcription factors—can be affected also through binding of CGs to steroid xenobiotic receptors [28]. In addition to direct binding to specific receptors, CGs can also function non-specifically. Thanks to their steroid nature, they can directly immerse in the cytoplasmic membrane and interact with lipids in this cellular compartment. Thereby, CGs can alter membrane fluidity, which was shown to be connected with downregulation of the interleukin-8 receptor and, thus, can be beneficial in, for example, inflammation treatment [29]. Inflammation, besides cardiac and cancer diseases, is another condition that can potentially be treated with CGs. The impact of CGs on the immune system and inflammation progression has been reviewed elsewhere, see reference [30]. The latter three conditions are, however, not the only ones in the therapeutic portfolio of CGs. The compounds have recently emerged also as potential antiviral agents, which, by various mechanisms, affect different steps of the viral life cycle.

## 3. Viral Life Cycle

The viral life cycle consists of several stages that have already been extensively reviewed elsewhere [31,32,33]. In brief, the cycle comprises three major steps: (1) viral entry, (2) replication, and (3) release of newly formed viral particles from the host cell. First, the virus finds a host cell using viral surface protein antigens, which bind receptors on the cell surface. These viral antigens are often glycosylated, which facilitates their shielding against the host immune system [34]. After the attachment to the cell receptors, the virus enters the cell. This step differs among enveloped and non-enveloped viruses. Both types of viruses, however, enter cells via receptor-mediated endocytosis, which is characterized by folding the plasma membrane in the intracellular space and forming endosomes encapsulating the viral particles. The formation of these endosomes is mediated by several proteins, the most common ones being clathrin, caveolin, and dynamin [35,36,37]. Once the endosomes are inside the cell, they mature into lysosomes, which decreases their inner pH, and as a consequence, the viral particles are released from the lysosomes either by fusion of the viral membrane with the lysosomal membrane (enveloped viruses) [38] or by the lysis of lysosomes via externalization of membrane-disrupting peptides (non-enveloped viruses) [39]. Besides endocytosis, enveloped viruses can get into cells directly via fusion of their membrane with the plasma membrane of the host cell, which is termed direct fusion [40], or by macropinocytosis, which is a specific type of endocytosis characterized by the nonspecific internalization of large amounts of extracellular fluid, solutes, and membrane in large vesicles, the macropinosomes [41]. Macropinocytosis is used mainly by larger viruses, such as murine amphotropic retrovirus [42] or the influenza virus [43]. After penetrating the host cell, the virus gets to the site of replication (trafficking) and exposes its genetic information (uncoating). Trafficking is realized via microtubules and dynein motors [44,45]. The target site for replication depends on the type of the virus. RNA viruses stay in the cytoplasm, except for *Orthomyxoviridae* and *Retroviridae*, whereas DNA viruses, except for *Poxviridae*, mostly enter the nucleus. Viruses can enter into the nucleus via nuclear pore complexes, which, in the case of smaller viruses, enable the whole capsid to pass through [46], whereas, in the case of larger viruses, the pores allow passing through the nuclear membrane only parts containing viral localization sequences [47]. In addition to the penetration through nuclear pores, some viruses can disrupt the nuclear membrane [48] or enter the nucleus during mitosis, when the nuclear membrane is naturally disrupted [49].

**Figure 2:**
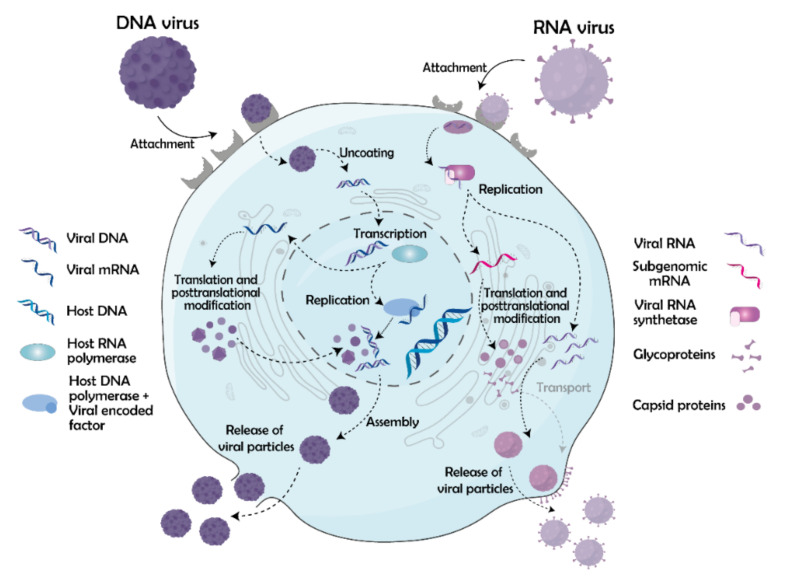
The life cycle of non-enveloped DNA and enveloped RNA viruses excluding retroviruses. (1) Life cycle of non-enveloped DNA viruses: a virus attaches to a specific cell surface receptor and enters the host cell. Subsequently, the virus exposes its DNA, which is transported to the cell nucleus and is replicated. Next, the DNA is transcribed into RNA followed by translation to viral proteins, which are then post-transcriptionally modified. The viral proteins and replicated DNA assemble into novel viral particles, which are then released from the host cells via disruption of the cell plasma membrane. (2) Life cycle of enveloped RNA viruses: a virus attaches to a specific cell surface receptor and enters the host cell (not indicated in the scheme) or inserts the RNA through the plasma membrane (indicated). This RNA is replicated via viral RNA synthetase and viral proteins are translated and post-transcriptionally modified. Next, the viral particles are released via exocytosis and are enveloped by the plasma membrane containing glycoproteins on its surface.

After reaching the site of replication, gene expression and synthesis of viral proteins and nucleic acids begins. This process is very well-known, and in general starts with the transcription of DNA into RNA. At first, a pre-initiation complex at the promoter sequence on the antisense strand of DNA needs to be formed. This preinitiation complex then binds to RNA polymerase, which synthesizes the new RNA. When the RNA polymerase reaches a specific sequence on DNA, called terminator, it detaches from the DNA and newly formed RNA undergoes post-transcriptional modifications: 5’-capping, 3’-cleavage, and polyadenylation, as well as subsequent splicing. The newly synthesized and modified RNA is then transported to the endoplasmic reticulum, where it undergoes the next step, translation, which takes place in the ribosomes of the rough endoplasmic reticulum [50]. For the correct function of ribosomes, the presence of potassium ions is essential as they help to stabilize the structure of ribosomal proteins, rRNAs, and also tRNAs [51]. A general mechanism of how genes and proteins are expressed is similar for all viruses since it is generally ensured by the cellular apparatus, but the exact replication process of viruses depends on their type based on the nucleic acid they include (Figure 2). A special case represents retroviruses, which use reverse transcriptase (RT) during replication. RT can be present both in DNA and RNA viruses. In the case of double-stranded DNA (dsDNA) viruses with RT, the cellular apparatus is used to produce RNA, which then serves as a template for the synthesis of DNA by RT. On the contrary, by positive-sense single-stranded RNA (ssRNA) viruses, RT synthesizes dsDNA from RNA in the cytoplasm. This dsDNA is then transported into the nucleus and incorporated into the host genome. The replication of all types of viruses is comprehensively reviewed in reference [52].

The last part of the viral life cycle, exit from cells, comprises three steps, assembly, release, and maturation. The assembly of the virus occurs after the synthesis of all viral components, in particular nucleic acids and capsid proteins, or all other necessary viral proteins involved in the viral life cycle. For example, in the case of the human immunodeficiency virus (HIV), those proteins comprise integrase, RT, protease, or matrix protein [53]. The particular process of the viral assembly depends on whether the capsid assembles simultaneously with the nucleic acid or not. The example of the first case is the avian infectious bursal disease virus, which uses ribonucleoproteins to form a scaffold for its dsRNA and facilitate the assembly of the whole capsid [54]. The other case is termed genome packaging and the nucleic acid is inserted into the capsid first after its full assembly via the so-called cis-acting element. This way is used for example by HIV [55]. Once the viral particle is assembled, it leaves the cell. Again, two major types of this process exist. In the case of non-enveloped viruses, the viral release is realized via cell lysis, which occurs in various modes. Some viruses, such as the simian virus 40, include specialized surface proteins, so-called viroporins, which can destabilize the plasma membrane [56]. Others, such as human adenovirus serum type 5, use the induction of autophagy to disrupt the host cell plasma membrane [57]. A different situation is in the case of enveloped viruses, which take along parts of the plasma membrane when leaving cells. This process is termed budding and is well-described for example on HIV [58] or herpes simplex virus (HSV) [59]. Besides the aforementioned main types of viral release, other mechanisms of this process exist; therefore, the listed herein are non-exhaustive. One of the recent studies by Ipinmoroti and Matthews reports on using exosome vehicles for the spreading of some viruses. These vehicles contain only parts of nucleic acids and viral protein co-receptors, which facilitate the infection of surrounding cells [60]. However, the viral particles are not infectious right after exiting cells. To become infectious, they have to maturate. Maturation is a process occurring outside cells and is characterized by changes in the specific arrangement of nucleic acids and proteins inside the viral capsid. Such changes are realized, for example, by viral protease (in the case of HIV-1), which cleaves group-specific antigen protein of partial components: a nucleocapsid, capsid, and matrix protein [61,62].

## 4. Effects of CGs on Viral Life Cycle 

CGs hamper the viral life cycle by their common mechanisms of action, mainly by disrupting the ion balance, autophagy of the host cell, or triggering various signaling cascades. The particular mechanisms of CG antiviral action differ for the particular viruses, but in general, CGs can disrupt both the early and late stages of the viral cycle. Currently, only viral attachment has not yet been described to be affected by CGs. Mostly they hamper viral entry and RNA processing. Moreover, viral protein synthesis is affected by CGs, since many essential enzymes for this process need a physiological intracellular K^+^ concentration in a host cell. In the following chapters, we will discuss the effects of CGs against particular viruses based on the life cycle phases they disrupt.

### 4.1. Disruption of the Early Stages of the Viral Life Cycle

**Figure 3:**
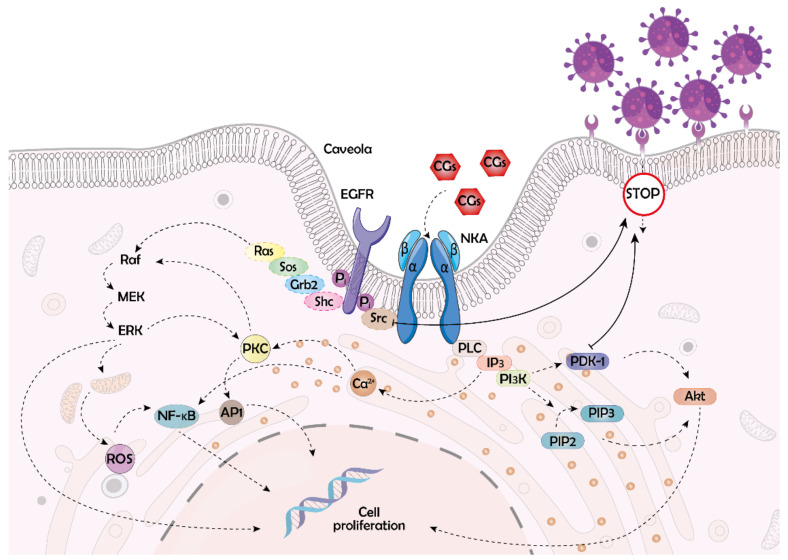
Molecular pathways triggered by binding of cardiac glycosides (CGs) to the α-subunit of Na^+^/K^+^-ATPase (NKA), which are involved in blocking the viral entry into host cells. By CG binding to NKA, conformational changes of its α-subunit trigger non-receptor tyrosine kinase (Src) to phosphorylate the epidermal growth factor receptor (EGFR). This leads to activation of Src homology 2 domain-containing transforming protein (Shc)/growth factor receptor-bound protein 2 (Grb2)/son of sevenless protein (Sos)/rat sarcoma protein (Ras) pathway. Ras further activates the serine/threonine kinase (Raf)/mitogen-activated protein kinase kinase (MEK)/extracellular signal-regulated kinase (ERK) pathway, which triggers reactive oxygen species (ROS) production activating nuclear factor kappa-light-chain-enhancer of activated B cells (NF-κB) and thereby affects the expression of genes in the nucleus and cell proliferation. The cell proliferation is affected also by protein kinase C (PKC), which activates transcription factors, such as AP1. PKC itself is activated either via the Ras/Raf/MEK/ERK pathway or by Ca^2+^ ions. The levels of Ca^2+^ can be elevated by another NKA-based pathway, the phospholipase C (PLC)/inositol triphosphate (IP_3_)/phosphatidylinositol-3 kinase (PI3K). The latter enzyme is responsible for the transformation of phosphatidylinositol-2-phosphate (PIP2) into phosphatidylinositol-3-phosphate. The PLC/IP3/PI3K pathway is responsible also for blocking the viral entry into cells, however, the exact mechanism of this process has been unclear, so far. The only involvement of 3-phosphoinositide-dependent protein kinase 1 (PDK1) has been proven. This enzyme physiologically activates protein kinase B (Akt), which then enters the cell nucleus and affects gene transcription. Besides, the viral entry into the host cells is blocked directly by Src, also with an unknown mechanism.

The earliest viral cycle step that has been described to be affected by CGs is the viral entry into a host cell. The mechanism by which CGs affect this process is mostly linked to triggering the non-receptor tyrosine kinase (c-Src) pathway. The interaction between c-Src and NKA has been extensively studied since the discovery of the NKA signaling function. It has been recently confirmed that c-Src directly interacts with the α1 subunit of the NKA [63]. When affected with compound **1**, tyrosine phosphorylation of c-Src is activated as a consequence of conformational changes on NKA. Phosphorylated c-Src then associates with EGFR and induces its phosphorylation. Both proteins then form a scaffold for further signaling cascade proteins, such as Src homology 2 domain-containing transforming protein 1, growth factor receptor-bound protein 2, son of sevenless protein, and ras. At the end of this complex signaling pathway, processes, such as the formation of reactive oxygen species (ROS) leading to apoptosis or autophagy, are triggered [30]. In particular, autophagy plays an important role in viral infections and, thus, its induction by CGs may be beneficial in the antiviral treatment [64]. Special attention must therefore be paid to CG’s ability to trigger molecular signaling pathways by binding the NKA α1 subunit (Figure 3).

The importance of the α1 subunit of NKA in Src-mediated viral entry was confirmed by Burkard et al. [65] for various coronaviruses, which are enveloped viruses with a positive-sense ssRNA genome causing mostly respiratory diseases. The authors showed that 50 and 10 nM concentrations of compound **1** and **2** (Figure 1), respectively, prevent the entry of Middle East respiratory syndrome, murine hepatitis virus, and feline infectious peritonitis virus in cervical cancer cells (HeLa) when treated 30 min. before infection and 2 h after the infection. The CG-prevented viral entry leads to the accumulation of virions close to the cell surface and reduced fusion with the plasma membrane. However, this can be rescued by the inhibition of the Src pathway. Burkard et al. [65] confirmed that combination treatment with 50 nM concentration of compound **1** and 50 nM Na^+^/K^+^-ATPase-mimetic Src-inhibitor peptide does not inhibit the viral entry in HeLa cells neither when affected 30 min. before infection nor 2 h after the infection. This demonstrates the involvement of the Src pathway in viral entry into cells. The authors speculate that the NKA-mediated c-Src signaling somehow interferes with clathrin-dependent endocytosis, which is the key process for the cellular uptake of the studied coronaviruses. The exact mechanism of such interference, however, remains unclear.

Similar to the discussed coronaviruses, the antiviral effect of CGs derived from specific NKA α1 subunit targeting has been reported also on the respiratory syncytial virus (RSV). Lingeman et al. [66] evaluated the antiviral activity of 25 nM and 25 µM concentration of compound **1** and **4** (a derivative of compound **3**, Figure 1), respectively, in lung carcinoma cells (A549) treated with the compounds 16 h before infection and observed blocked tyrosine-phosphorylation of EGFR, which is otherwise triggered by RSV. As a consequence, the entry of RSV into host cells is significantly decreased. In the case of compound **1**, the virus yield was reduced by 3 log units in comparison to non-treated cells. This confirms the important role of the NKA-Src-EGFR pathway in this early process of the viral life cycle. In addition to Src signaling, another pathway is involved in the antiviral activity of CGs. Namely, the phosphatidylinositol 3-kinase/3phosphoinositide-dependent protein kinase 1/p90 ribosomal S6 kinase (PI3K/PDK1/RSK2) pathway, which is involved in compound **1** action against transmissible gastroenteritis coronavirus (TGEV). In contrast to the latter study, inhibition of Src by 0.2 µM compound **1** (1 h pre-treatment before infection and 6 h treatment after the infection) does not impair the anti-TGEV activity in swine testicular cells (ST), as confirmed by Yang et al. [67]. On the contrary, only inhibition of PDK1 significantly decreases the antiviral potency of compound **1**. Given the fact that NKA can trigger PI3K/PDK1 pathway, also independently on Src, the latter result is not contradictory. The differences among the coronaviruses are most probably caused by their impact on the expression levels of NKA α1 subunits in host cells. 

Surprisingly, Yang et al. [68] recently reported also an NKA-independent mechanism of the antiviral action of CGs against coronaviruses. It is based on Janus kinase 1 (JAK1) proteolysis and downregulation. Observations in swine testis cells infected with TGEV and human coronavirus OC43 confirmed that this pathway is independent of NKA and also on PI3K/PDK1/RSK2 pathway, which is triggered simultaneously with the JAK1 downregulation. The proteolysis of JAK1 is caused by the activation of neural precursor cell expressed developmentally downregulated protein 4 E3 ubiquitin-protein ligase family interacting protein 1/2. This process, which is activated by compound **1** (250 nM, 5 h of treatment), also occurs in uninfected cells and must be mediated by some membrane protein distinct from NKA. This has been shown in experiments with compound **5** (Figure 1), which, even in an impermeable form of a bovine serum albumin-conjugate, also activates the discussed process and, thus, suppresses the life cycle of coronaviruses. The potency of CGs against coronaviruses might be useful also in the recent pandemics of the severe acute respiratory syndrome coronavirus 2 (SARS-CoV-2), causing coronavirus disease-19 (COVID-19). Many studies have been published on this topic showing that CGs could be beneficial in COVID-19 treatment. They appear to be effective both in the prophylactic and therapeutic treatment, which suggests that they can affect different stages of the SARS-CoV-2 life cycle. [69,70]. According to the urgent need for anti-Covid-19 medicine, the published studies focused rather on identifying the active compounds than on elucidating their mechanisms of action. Most probably, it will not be much different from that reported on other coronaviruses. This has been suggested as well by Souza et al. [71], in a review article focused on CG action against SARS-CoV-2. The most probable mechanism of the anti-Covid-19 action of CGs is the inhibition of the nuclear factor kappa B (NF-ĸB) through Src signaling linked to NKA. Even though pivotal experiments show good tolerability *in vivo* after 7 days of daily administration of 25 µL of *Nerium oleander* extract containing 130 µg·mL^−1^ of compound **6 (**Figure 1) in golden Syrian hamster COVID-19 models [72], much more data must be collected before CGs could be evaluated clinically in the treatment of coronaviruses and particularly the SARS-CoV-2.

Coronaviruses are not the only viruses, the life cycle of which is inhibited in their early stages. Recently, several drug screening studies have been published presenting the identification of drug candidates for the treatment of the Ebola virus (EBOV). EBOV belongs to the *Filoviridae* family and contains negative-sense ssRNA. It causes severe hemorrhagic fever and can rapidly spread and lead to large epidemics. Unfortunately, there is currently no approved treatment against this virus. As reported in the screening studies, various CGs could be potentially useful against EBOV. The first one, published by Edwards et al. [73] in 2015, showed compounds **7**, **8**, and **9** (Figure 1) as potent inhibitors of EBOV. Unfortunately, their half-maximal antiviral inhibitory concentrations (IC_50_) were lower than the half-maximal cytotoxic concentrations (CC_50_) after 24 h in human embryonic kidney cells (HEK 293T), which disadvantages them for potential clinical use. Moreover, compound **1** was evaluated as an EBOV inhibitor, as presented by Dowall et al. [74]. Similar to the aforementioned CGs, also compound **1** shows good anti-EBOV activity in human lung fibroblasts (MRC-5) and Vero E6 cells by reducing viral RNA levels but causes significant damage to the cells at the effective concentrations (>20 nM) after 3 days of treatment. In addition to the latter study, Du et al. [75] recently reported 250 nM compounds **1**, **5**, **7**, and **9** to inhibit EBOV entry into host HeLa cells when administered 8 h before infection and incubated for further 3 days after the infection. The authors suggested this effect could be connected with the Ca^2+^ ion imbalance caused by the studied CGs. They identified the two-pore segment channel 2, which mediates Ca^2+^ release from lysosomes, to play an important role in EBOV entry into host cells. The physiological balance of Ca^2+^ ions is important for the EBOV entry; however, the exact mechanisms of this process have not been fully revealed, yet. Importantly, CGs are effective against EBOV, though, a balance between the antiviral and cytotoxic activity must be sought.

In addition to EBOV, another example of a potential CG target is human cytomegalovirus (HCMV). HCMV is an enveloped dsDNA virus from the *Betaherpesvirinae* family, which dominantly causes latent infection, but can be dangerous in immunosuppressive patients [76]. Kapoor et al. [77] reported decreased levels of immediate–early, early, and late HCMV proteins after treatment of human foreskin fibroblasts (HFFs) with compounds **1** and **5** at concentrations of 0.025–0.1 µM either 1 h before or simultaneously with the infection of host cells with HCMV and incubated for 3 days. The antiviral effect of CGs occurs early after the viral attachment to the host cells but before DNA replication, since pretreatment with CGs does not decrease viral yields more than when cells are treated with CGs at the time of infection. Thus, also, in this case, the viral entry into the host cells might be impaired. The authors connect this antiviral activity of CGs with their ability to downregulate the expression of the human ether-à-go-go-related gene (hERG), which encodes the K^+^ ion channel. The hERG downregulation has been described already in the studies on the anticancer potential of CGs [78], because the overexpression of hERG often occurs in cancer cells [79] and, as reported by Kapoor et al. [77] also after HCMV infection. Downregulation of hERG expression by CGs leads to a physiological state since, in healthy cells, hERG is commonly not expressed [77].

Besides hERG, viruses are well-known to interact with various ion channels which makes them an interesting therapeutic target [80]. Therefore, the involvement of CGs in antiviral therapy against HCMV could be an effective option. Cai et al. [81] further studied the interaction of different analogs of compound **9** with HCMV and tried to modify their structure to increase their antiviral activity. In the structure–activity relationship studies on HFFs, they showed that this can be ensured by changing D-sugars to L-sugars and shortening the oligosaccharide chain. The authors connected this result with an altered affinity of the studied derivatives to different subunits of NKA, suggesting furthermore complex studies of the relationship between CGs and NKA in cells affected by HCMV. In addition to compound **9**, also compound **10** (Figure 1) and its analogs have been studied concerning HCMV. According to Cohen et al. [82], compound **10** (12.5–50 nM, 24 h) acts via limiting methionine supply in MRC-5 cells. Methionine gets into cells via co-transport with Na^+^ ions, concentrations of which are affected by compound **10**-NKA interaction. By reducing the pool of methionine in cells, compound **10** causes decreased expression of viral intermediate early genes. Such activity is independent of the signaling function of NKA, thus, the ion imbalance is the only mechanism of action of compound **10** against HCMV.

Among the viruses, which are affected by CGs in the early stages of their life cycle, there is also HSV, which is an enveloped dsDNA virus falling into the *Herpesviridae* family [83]. HSV can develop a latent infection in the nervous system and after reactivation cause life-threatening conditions. Dodson et al. [84] were the first to report the ability of compound **1** to reduce the expression of HSV type 1 (HSV-1) immediate–early and early genes (full inhibition of the formation of HSV-1 plaques under >100 nM compound **1** in Vero cells after 72 h). Next, Su et al. [85] evaluated compound **9** in HSV-1-infected Vero cells and reported the IC_50_ of compound **9** to be equal to 0.05 µM (CC_50_ = 10.66 µM). Comparing antiviral and cytotoxic potency, the selectivity index of compound **9** is equal to 213.2 showing potent anti-HSV activity. Compound **9** reduces viral DNA synthesis and decreases mRNA levels of the early gene *UL52* coding DNA primase. In addition to compound **9**, compounds **1** and **5** have also been tested and it has been confirmed that the glycone moiety is necessary for the antiviral activity. This points to the essential role of NKA in the anti-HSV action of CGs since the glycone moiety has previously been confirmed to be crucial for a stable block of the NKA function [86].

Besides HSV, CGs might be useful also against the chikungunya virus (CHIKV), the life cycle of which is inhibited by these compounds at the early stages. CHIKV is a mosquito-borne enveloped positive-sense ssRNA virus from the *Togaviridae* family, occurring mainly in tropical and subtropical regions of Africa, in the Indian Ocean islands, South and Southeastern Asia, and across several Pacific nations. It causes fever, headache, myalgia, rash, and joint pain, which may lead to a bent or stooping posture [87]. Ashbrook et al. [88] demonstrated in human cells from osteosarcoma (U-2 OS) that the inhibition of NKA by 1 µM compound **5** administered 1 h before the infection causes the post-entry block of CHIKV infection. They proposed that this might be a consequence of altered ion levels inside cells since alphavirus particle maturation is sensitive to the ionic strength of the medium when cultured *in vitro*. Moreover, some alphavirus proteins, particularly 6K protein, function as ion channels selective for Na^+^, K^+^, and Ca^2+^ ions and ensure the ion concentrations needed for the proper process of particle maturation. The levels of the crucial ions are directly affected by the function of NKA, which might have an inhibitory impact on viral protein functioning. Unfortunately, the exact mechanism of action has not been fully explored, yet.

Similar effects of CGs have been observed also in other tropical viruses. The disease caused by CHIKV is often misdiagnosed as dengue. Dengue virus (DENGV) is also an enveloped mosquito-borne, positive-sense ssRNA virus, which belongs to the *Flaviviridae* family and is known for causing severe disease with high mortality rates. A mild form is characterized by similar symptoms as CHIKV disease, the severe dengue disease manifests itself by a strong hemorrhagic fever and a life-threatening condition called dengue shock syndrome. Cheung et al. [89] studied the anti-DENGV effects of compound **7** and observed a dose-dependent antiviral effect, with the highest inhibition rates (80–100%) at 1 µM concentration for post-infection treatment in human hepatocellular carcinoma cells (Huh-7) and human umbilical vein endothelial cells. The inhibitory effects occur only when compound **7** is used 24 to 48 h after infection of cells with DENGV, which means that this CG affects most probably viral RNA synthesis.

### 4.2. Disruption of RNA Synthesis and Processing

RNA synthesis is one of the viral life cycle phases, which is affected by the unspecific actions of CGs. Norris et al. [90] suggested that the RNA synthesis is dependent on precise intracellular concentrations of Na^+^ and K^+^ ions. They studied the anti-RSV activity of compounds **5** and **9**, as well as that of ionophore antibiotics salinomycin, valinomycin, and monensin in human epithelial cells (HEp-2). The strongest antiviral effect (80% inhibition of RSV replication) exerts compound **5** (260 nM), compound **9** (260 nM), and valinomycin (32 nM) when used 2–4 h post-infection, indicating that the compounds most probably interfere with the synthesis of viral RNA. Given the commonly known mechanism of antibiotic action, it is clear that the role of K^+^ and Na^+^ ions in RNA synthesis is irreplaceable. Currently, it is well-known that ion channels are good targets for antiviral therapies since different ions are important throughout the whole viral cycle, not only during RNA synthesis [91,92,93]. Some of the viral proteins even function as ion channels themselves [94]. Therefore, it seems logical to think of NKA as the optimal target for antiviral therapies and consider CGs as ideal antiviral agents.

The evidence from the literature speaks clear: CGs are emerging in antiviral therapies. However, considering RNA synthesis, not much evidence has been published showing CGs intervention in this process. Besides the aforementioned study of Norris et al. [90], Guo et al. [95] reported on CGs acting against Zika virus (ZIKV). ZIKV is an enveloped flavivirus containing positive-sense ssRNA, for which there is currently no approved treatment. The authors reported that compounds **1** and **5** potently inhibit ZIKV infection in Vero, Huh-7, and human glioblastoma U251 cells with 4–5 log unit reductions of viral particle production at 784 nM concentration and 48 h of treatment. They also proved that compounds **1** and **5** inhibit ZIKV RNA synthesis through the blockade of NKA. Moreover, the authors showed that both CGs are beneficial also *in vivo* and that compound **1** decreases viral loads in mouse brains (mice treated with 2 mg·kg^−1^ of compound **1** once per day for 5 days) and prevents ZIKV-mediated brain injuries. The latter two studies are the only ones to report inhibited viral RNA synthesis under the treatment of infected cells with CGs. However, much more data have been published on the effects of CGs against viral RNA processing.

Intervention into RNA processing is one of the most commonly described mechanisms of the antiviral activity of CGs. The effects of CGs on RNA splicing have been extensively studied and partial mechanisms of such process have been revealed. RNA splicing is a posttranscriptional modification, which enables the creation of multiple functional RNAs from the primary transcript. Splicing can be (i) constitutional, upon which introns are removed and exons are linked together to form a mature mRNA, and (ii) alternative, which consists of diverse exon combinations so that a diverse RNA palette is created, which ensures broader protein diversity [96]. It is exactly the alternative splicing machinery, which is used by viruses to produce their mRNAs. Both RNA and DNA viruses are extremely dependent on alternative splicing processes in cells [97]. As reported by Wong et al. [98], compound **5** can suppress HIV-1 replication by altering viral RNA splicing (90% inhibitory concentration IC_90_ equal to 25 nM after 8 days in peripheral blood mononuclear cells; PMBCs). The nature of such action lies in affecting the cyclin-dependent kinase 2-like kinase family of serine-arginine (SR) protein kinases, thereby modifying several SR proteins, such as SR protein 2 and transformer 2 protein homolog beta, which promote the use of splice sites of an HIV pre-mRNA transcript. By altering the structure of these proteins, compound **5** induces over-splicing of HIV RNA, which results in reduced accumulation of viral mRNAs encoding structural proteins for assembly of new virions. Moreover, compound **5** downregulates the expression of regulator of virion protein expression by altering the specific pre-mRNA splicing site for this regulator. Moreover, compound **5** potently suppressed the replication of HIV-1 clinical strain R5 BaL *in vitro*. In HeLa cells, compound **5** exerts this suppressive activity at concentrations of hundreds of nM. This is unfortunately much higher than clinically used concentrations (max. 5 nM), however, in patient’s peripheral blood mononuclear cells (PBMCs), Wong et al. [98] observed strong suppression of HIV replication even at a 2 nM concentration of compound **5** when treated for 21 days. The differences in antiviral action of compound **5** against HIV-1 in HeLa and PBMCs might be caused by different production of NKA in these cells and alterations in the affected pathways in cancerous cells distinct from noncancerous ones. In general, compound **5** is a potent inhibitor of HIV replication, and elucidation of the exact mechanism of its antiviral action as well as further *in vitro* and *in vivo* studies are desirable.

However, compound **5** is not the only CG, the anti-HIV activity of which has been reported. Wong et al. [99] brought evidence that twelve other CGs act in a similar manner as compound **5**, which means that they can suppress the expression of HIV proteins and inhibit replication of this virus. Structurally, aglycones (compound **3** and **11**) and compound **7** were 4–11-fold less potent against HIV in infected T-cells from clinical patients than compound **5** after 21 days. Compounds **1**, **2**, **9**, **10**, and **12** (Figure 1), and a semisynthetic derivative RIDK-34 (compound **13**, Figure 1) exert 2–8-fold higher anti-HIV effectivity in comparison to compound **5** when tested in HeLa cells, all being active at IC_90_ of max. 25 nM. For example, compound **9** requires even 15–26-fold lower concentration for HIV inhibition in PBMCs than it is recommended for clinical treatment of patients with heart diseases. Surprisingly, minor changes in the structure of the CG steroid cores cause big differences in the anti-HIV potency. This suggests that the mechanism, by which the compounds suppress HIV replication is highly specialized. Wong et al. [99] presumed according to their experiments that multiple NKA-based signaling pathways are involved in HIV replication. Mainly it is MEK1/2-ERK1/2 signaling, the inhibition of which was the only way, how to restore HIV gene expression in CG-treated cells. However, it seems that the whole NKA signaling machinery is involved in HIV-1 replication since activation of the Src-EGFR-Ras-Raf-MEK1/2-ERK1/2 pathway contributes to the suppression of this process by CGs. Wong et al. revealed large, but still only partial, evidence for an important role of CGs in HIV treatment. Moreover, further CGs, such as compound **6** [100] or CGs from *Elaeodendron croceum* [101] are active anti-HIV compounds. Given the low concentrations needed and a large palette of active compounds, the family of CGs represents a very promising anti-HIV option and it is worth studying their activity further.

**Figure 4:**
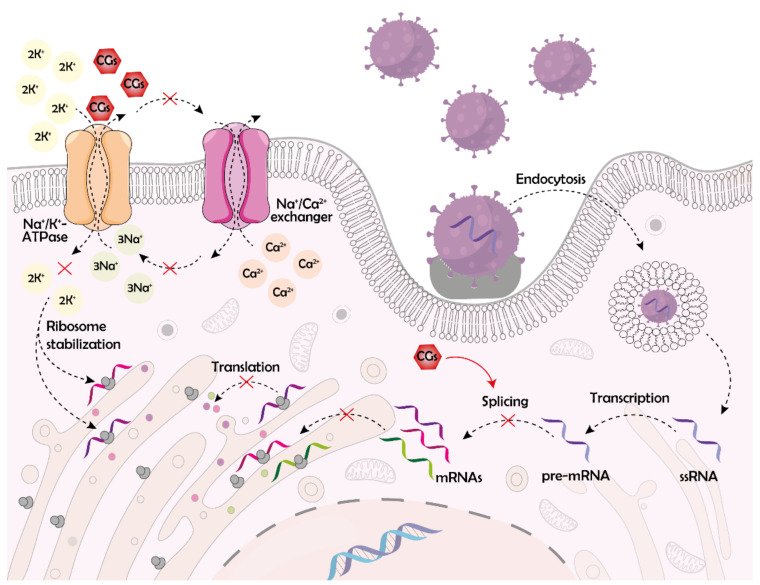
The effect of cardiac glycosides (CGs) on viral RNA synthesis and processing, and protein translation. CGs block RNA splicing by further unexplained mechanisms, which are likely linked to the Na^+^/K^+^-ATPase (NKA) signaling function (not indicated in the scheme). The result of this is a decreased pool of viral messenger ribonucleic acids (mRNAs) and a subsequent block of viral protein synthesis. The translation of viral proteins is hampered also by blocking the pumping function of NKA. As a consequence of this, the intracellular level of K^+^ ions is decreased, which negatively affects ribosomal function. K^+^ ions play a physiologically important role in the stabilization of the ribosomal complex. The improper function of ribosomes caused by K^+^ depletion leads to decreased translation of viral proteins.

Besides HIV, CGs potently disrupt RNA splicing of adenoviruses, which are non-enveloped dsDNA viruses causing predominantly mild respiratory diseases but are life-threatening in immunosuppressed patients. The first evidence for the anti-adenovirus activity of compound **5** was published by Hartley et al. [102] in 2006, who connected this action with the ion imbalance caused by CGs. They observed a significant difference in the antiviral potency of compound **5** against DNA and RNA viruses. While plaque formation of human encephalomyocarditis virus (RNA virus) was not inhibited at all in hamster kidney fibroblasts (BHK-21) after 7 days of treatment with the highest used concentration of 60 ng·mL^−1^ of compound **5**, DNA viruses were significantly affected by this compound. HSV type 2 (HSV-2; tested in MRC-5, Vero, and BHK-21 cells), HSV-1 (in Vero cells), HCMV (in MRC-5 cells), varicella-zoster virus (in MRC-5 cells), and adenovirus (in A549 cells) were inhibited by compound **5** in plaque inhibition assay at IC_50_ ranging from 20–77 ng·mL^−1^ after 48 h for HSV and 7 days for other viruses. This suggests that the specific mechanism of action might lie in transcription and RNA processing. Such a hypothesis has been proven by Grosso et al. [103] in 2017. They first confirmed the results of the preceding study, observing the inhibited genome replication after treatment of A549 cells 1 h post-infection with compounds **5**, **9**, and **13** at concentrations of hundreds on nM for 24 h. The authors reported also significantly inhibited expression of a delayed early protein E4orf6 and major late capsid protein, hexon. The mechanism of action probably lies in the alteration of RNA splicing during the early phases of infection. The compounds also block the transition from 12S and 13S RNA to 9S RNA at the late stages of the virus replication, which shows a large complexity of the antiviral action of CGs. The most potent compound tested by Grosso was compound **13**, which achieves the same viral inhibition (3.5 log reduction of virus yield) as compound **5** already at approximately 12-fold lower concentration (12.5 nM). Interestingly, the antiviral effect lasts only in a constant presence of the compounds and is rapidly reversible. In general, Grosso et al. confirmed the potentially very useful antiviral activity of CGs connected with RNA processing (Figure 4).

### 4.3. Disruption of Viral Protein Synthesis and Release

RNA processing is perhaps the most described viral life cycle phase inhibited by CGs. However, these compounds can intervene also in the following step, protein synthesis. Recently, CGs have been reported to inhibit the translational machinery of the influenza virus. Influenza viruses are enveloped negative-sense ssRNA viruses from the *Orthomyxoviridae* family causing seasonal respiratory disease epidemics [104]. The activity of CGs against this virus has been reported by Amarelle et al. [105] in 2019. They found out that minimal doses, i.e., 20, 50, and 100 nM, of compounds **1**, **5**, and **12**, respectively, inhibit influenza A virus (IAV) in A549 cells 24 h after infection by decreasing K^+^ concentration and blocking viral protein synthesis independently on other NKA-related mechanisms, such as triggering signaling pathways. Interestingly, 24 h treatment with 20 nM compound **1**, which does not cause cytotoxicity, is sufficient to prevent the viral replication for subsequent 48 h. Moreover, compound **1** (50 µg·kg^−1^ per day for 2 days) delays mortality of CG-sensitive mice infected with a lethal dose of IAV and decreases viral titers in their lungs. Besides direct antiviral effects, CGs may play their role also in suppressing IAV-related cytokine storm, which is a complicating condition linked to immune system overreaction. A severe cytokine storm can lead also to multiorgan failure and death. Via inhibition of NF-ĸB signaling, CGs can block cytokine expression and the emergence of the cytokine storm. Pollard et al. [106] studied the ability of compound **9** (0–30 μg per 100 g per day for 4 days) to inhibit cytokine storms in IAV-treated cotton rats. They observed suppressed levels of tumor necrosis factor α, human growth-regulated oncogene/keratinocyte chemoattractant, macrophage inflammatory protein-2, monocyte chemoattractant protein-1, and interferon γ in rat lungs after the 4-day treatment with compound **5**. Since cytokine storm occurs also after infection with other respiratory viruses, such as coronaviruses, the authors suggest the use of compound **9** also for example for the treatment of COVID-19 disease, as discussed before.

Besides IAV, the inhibition of protein synthesis caused by CGs has been described also for HSV. A pivotal study on the anti-HSV activity of CGs was performed by Hartley et al. [102] in 2006. In this aforementioned study, compound **5** potently suppressed the infection of HSV, but this activity could be reversed by adding 20 mM K^+^. This suggests that the nature of the antiviral effect of compound **5** is again the ion imbalance. K^+^ ions are required for the proper function of various enzymes, also of these involved in viral protein synthesis. Indeed, Bertol et al. [107] described another CG compound **14** (Figure 1) to fully inhibit HSV protein synthesis in Vero cells through interaction with NKA and decreasing the intracellular K^+^ concentration (IC_50_ after 48 for HSV-2 equal to 0.04 µM, after 72 h for HSV-1 equal to 0.13 µM and 0.06 µM for KOS strain and 29R strain respectively). Besides compound **14**, the anti-HSV activity has been reported also for a related compound **15** (Figure 1), which is present in *Carissa spinarum*. Wangteeraprasert et al. [108] reported its activity in Vero cells with IC_50_ being 120.2 and 168.2 µM for HSV-1 and HSV-2, respectively, (1 h post-infection treatment, cultivation for 5 days), which is much higher than for commonly used acyclovir (IC_50_ = 2.8 µM). Unfortunately, the authors did not study the mechanism of action further. The most recent studies regarding CGs in the anti-HSV research have been presented by the group of Boff et al. [109]. They reported 16 novel derivatives of compound **3**, two of which, compound **16** (Figure 1) and compound **17** (Figure 1) have been identified as potent HSV inhibitors. Their IC_50_ after 48 h (Vero cells treated 1 h post-infection) are equal to 0.23 and 0.24 µM against HSV-1 (KOS strain); 0.18 and 0.19 µM against HSV-1 (29-R strain); and 0.27 and 0.30 µM against HSV-2 (333 strain) for compound **16** and **17**, respectively. The mechanism of action of the compounds seems to be complex. In Vero cells, Boff et al. [110] reported both compounds (at concentrations of 1/4 to 1 × IC_50_; 18 h of incubation) to reduce the expression levels of the *UL42* gene encoding DNA polymerase processivity factor, and *gD* gene encoding envelope glycoprotein D precursor. CGs, thus, interfere with DNA and protein synthesis. Moreover, treatment with CGs leads to decreased levels of ICP27 protein, which is generally involved in gene expression. Unfortunately, the authors did not study the origin of such observations and only speculated on the mechanisms of action.

The latter two novel compound **3** derivatives have been described to inhibit also viral release from host cells. This process is the last step of the viral life cycle, which has been described to be potentially targetable with CGs. Boff et al. [110] suggested that their derivatives of compound **3**, i.e., compounds **16** and **17**, inhibit viral release from Vero cells based on measuring intracellular and extracellular titers of HSV (95% reduction in viral extracellular yields after 24 h of incubation at the above-discussed IC_50_ of both compounds). Moreover, they reported reduced areas of the formed viral plaques, which shows that cell-to-cell spreading is inhibited. This points again to the inhibition of viral release. Moreover, a previous study by Su et al. [85] suggested that CGs might inhibit viral release. In this study, compound **9** was compared in HSV-infected Vero cells to acyclovir, which interferes with the early stages of the viral life cycle. When the cells were treated with a 6 µM concentration of acyclovir for 24 h, no significant difference between intracellular and extracellular HSV titers could be observed, while after 24 h treatment with 50 nM compound **9**, the extracellular levels of HSV were decreased by 96%. Unfortunately, none of the studies elucidated the mechanism by which CGs block the viral release. The basis for such activity, thus, remains unclear. Nevertheless, CGs by complex mechanisms hamper the viral life cycle and it is clear that their future in the field of antiviral therapies is promising.

## 5. Conclusions

CGs, originally developed for heart conditions, currently represent highly promising multifunctional compounds [111,112]. Although clinically they are still used exclusively for the treatment of cardiac conditions, in research and clinical trials, they have greatly emerged as anticancer and also antiviral drugs. The current knowledge on CG antiviral properties is mostly based on pivotal observations, which widely differ depending on the particular CG and virus. The reported antiviral mechanisms of CGs involve both unspecific and highly specific actions. The unspecific antiviral mechanism is connected with disrupting the ion balance in cells by CG blocking the pumping function of NKA. The specific action against viruses is, then, derived mainly from the interaction of CGs with the α1 subunit of NKA. This enzyme is a key member of a large signaling cascade connected with Src, EGFR, and Ras/Raf/MEK/ERK pathways, which are crucial for the intracellular entry of several viruses, such as adenoviruses. By activating Src kinase, as well as triggering the PI3K/PDK1/RSK2 pathway, CGs potently hamper the entry of the viral particles into cells. The entry of the virus into cells might be blocked by CGs even via the NKA-independent JAK1 pathway, which shows that the mechanisms of antiviral action of CGs are complex. As we showed here, CGs can interfere with any step of the viral life cycle except for the viral attachment to a host cell. For each virus, different mechanisms of action have been proposed and, thus additional studies comparing the effects among individual viral types are needed to understand the mechanisms properly. In this review article, we aimed to show evidence of CGs as antiviral agents, and inspire researchers to focus their efforts on these compounds.

## Data Availability

Not applicable.

## Abbreviations

A549	lung carcinoma cells
AP1	transcription factor
CC_50_	half-maximal cytotoxic concentration
CG	cardiac glycoside
Covid-19	coronavirus disease-19
DENGV	dengue virus
dsDNA	double-stranded deoxyribonucleic acid
dsRNA	double-stranded ribonucleic acid
EBOV	Ebola virus
EGFR	epidermal growth factor receptor
E4orf6	delayed early adenoviral protein
ERK	extracellular signal-regulated kinase
Grb2	growth factor receptor-bound protein 2
HCMV	human cytomegalovirus
HEK293T	human embryonic kidney cells
HeLa	cervical cancer cell line
Hep-2	human epithelial cells
hERG	ether-à-go-go-related gene
HFFs	human foreskin fibroblasts
HIV	human immunodeficiency virus
HIV-1	human immunodeficiency virus type 1
HSV	herpes simplex virus
HSV-1	herpes simplex virus type 1
HSV-2	herpes simplex virus type 2
Huh-7	human hepatocellular carcinoma cell line
CHIKV	chikungunya virus
IAV	influenza A virus
IC_50_	half-maximal antiviral inhibitory concentrations
IC_90_	90% antiviral inhibitory activity
JAK1	janus kinase 1
MEK	mitogen-activated protein kinase kinase
NF-ĸB	nuclear factor kappa B
NKA	Na^+^/K^+^-ATPase
PBMC	peripheral blood mononuclear cell
PDK1	3-phosphoinositide dependent protein kinase 1
PI3K	phosphatidylinositol 3-kinase
Raf	rapidly accelerated fibrosarcoma protein
Ras	rat sarcoma protein
ROS	reactive oxygen species
RSK2	p90 ribosomal S6 kinase
RSV	respiratory syncytial virus
RT	reverse transcriptase
SARS-CoV-2	severe acute respiratory syndrome coronavirus 2
Shc	Src homology 2 domain-containing transforming protein
Sos	son of sevenless protein
SR	serine-arginine
Src	non-receptor tyrosine kinase
ST	swine testicular cells
ssRNA	single-stranded ribonucleic acid
TGEV	transmissible gastroenteritis coronavirus
U251	human glioblastoma cells
U-2 OS	human osteosarcoma cells
UL52	DNA primase
ZIKV	Zika virus
